# Genetic variants of *ABCB1* and *CES1* genes on dabigatran metabolism in the Kazakh population 

**DOI:** 10.22088/cjim.15.3.499

**Published:** 2024-08-01

**Authors:** Ayan Abdrakhmanov, Elena Zholdybayeva, Aizhana Shaimerdinova, Gulmira Kulmambetova, Svetlana Abildinova, Rustam Albayev, Gulnara Tuyakova, Elena Rib, Zhanasyl Suleimen, Zhanar Abdrakhmanova, Makhabbat Bekbossynova

**Affiliations:** 1 Department of Arrhythmology, National Research Cardiac Surgery Center, Turan 38, Astana, Kazakhstan; 2 Hospital of the Medical Center of the Office of the President, E 495, Astana, Kazakhstan; 3 National Center for Biotechnology, 13/5, Kurgalzhynskoye road, Astana, Kazakhstan; 4 Medical University Astana, Beibitshilik St 49/A, Astana, Kazakhstan

**Keywords:** CES1, ABCB1, Genetic polymorphisms, Dabigatran, Pharmacogenetics, Atrial fibrillation

## Abstract

**Background:**

Allelic variants of genes encoding enzymes of the esterase system (*CES1*) and P-glycoprotein (*ABCB1*) can change the metabolism and pharmacokinetics of dabigatran. Therefore, they act as determining factors in the development of side effects, especially bleeding. We analyzed the genotype–phenotype relationship of *ABCB1* (rs1045642, rs4148738, rs2032582, and rs1128503) and *CES1* (rs8192935, rs71647871, and rs2244613) polymorphisms in patients with atrial fibrillation who had been treated with dabigatran.

**Methods:**

A total of 150 patients were recruited for this study. TaqMan technology was used for SNP genotyping.

**Results:**

Patients with the rs2244613 GG genotype had a lower concentration (55.27 ± 34.22 ng/ml) compared to those with the TT genotype (63.33 ± 52.25 ng/ml) (additive model, P = 0.000). Individuals with the rs8192935 AA genotype had a lower concentration (52.72 ± 30.45 ng/ml) compared to those with the GG genotype (79.78 ± 57 ng/ml) (additive model, P = 0.001). The APTT values among the different genotypes of the *ABCB1* SNPs, rs4148738 and rs1045642, were significantly different (P = 0.035 and P = 0.024, respectively).

**Conclusion:**

Our research demonstrates that the *CES1* polymorphisms, rs8192935 and rs2244613, are associated with the pharmacodynamics and pharmacokinetics of dabigatran in the Kazakh subpopulation.

Atrial fibrillation (AF) is a fast, irregular atrial rhythm disorder requiring anticoagulant therapy owing to its high risk of causing strokes and systemic thromboembolic complications (1). Reducing the risk of thromboembolic complications and ensuring the effective use of anticoagulant therapy in patients with non-valvular AF are urgent medical and social problems in modern society and healthcare, both in the Republic of Kazakhstan and worldwide. Direct oral anticoagulants act as first-line drugs and are used for thromboembolic complications in non-valvular AF (rivaroxaban, dabigatran etexilate, and apixaban) (2–4).

Dabigatran etexilate is a non-pharmacologically active prodrug that inhibits coagulation factor IIa (thrombin). Following oral administration, dabigatran etexilate is rapidly absorbed in the gastrointestinal tract and converted to dabigatran in the liver and plasma via hydrolysis, which is catalyzed by the hepatic carboxylesterase, *CES1*, playing a vital role in the conversion of the prodrug to its active state (5–8). Dabigatran’s half-life is 12–17 h and it has a bioavailability of 35%. It is predominantly excreted unchanged by the kidneys (approximately 80%) (5, 7, 9). The drug is prescribed at a fixed dose of 110 or 150 mg twice daily (10). 

In the RE-LY study (11), dabigatran (150 mg) treatment twice daily reduced the risk of stroke and systemic embolism by 35% compared with that of warfarin treatment and decreased the risk of major bleeding by 20%, thus supporting its effectiveness at preventing thromboembolic complications. Although dabigatran, like other oral anticoagulants, does not require routine monitoring, interindividual differences in plasma concentrations of the drug have been identified. Based on early studies, the plasma concentration of dabigatran depends on factors such as age, renal function, sex, and weight (11).

The prodrug, dabigatran etexilate, is a substrate for the P-glycoprotein transporter; hence, its interaction with P-gp inhibitors can increase the level of dabigatran in the blood and lead to bleeding. Paré et.al. (8) conducted genome-wide association studies in a randomized evaluation of long-term anticoagulant therapy (RE-LY) in 2,944 participants. Their analysis identified several common genetic variants at the *CES1* and *ABCB1* loci that correlated with trough and peak levels of dabigatran. According to their data, allelic variants of genes encoding enzymes of the esterase system (*CES1* and *CES2*) and P-glycoprotein (*ABCB1*) are the most promising for studying the effect of genetic predisposition on the pharmacokinetics of direct oral anticoagulants. These alleles can change the metabolism and pharmacokinetics of drugs and, therefore, act as a determining factor in the development of side effects, especially bleeding (11). The most promising genetic variants for *CES1* are the polymorphisms, rs8192935, rs2244613, and rs71647871, and those for *ABCB1* are rs4148738, rs1045642, rs2032582, and rs1128503 (7, 10, 12). Studies in various ethnic groups have demonstrated pronounced interracial and interethnic differences in drug sensitivity, which may affect the choice of pharmacotherapy strategy and personalization of drug dosing regimens. 

For example, in the Chinese population, the frequencies of *CES1* rs2244613 and rs8192935 alleles are significantly different from those in European populations, but those of *ABCB1* rs1045642 and rs4148738 alleles were similar to European ones (13, 14). Previous studies showed that the allele frequencies of rs4148738, rs1045642, rs2032582, and rs1128503 of *ABCB1* in different populations do not differ significantly (15). The work of Sychev et al. (16) showed that the frequencies of rs2244613 alleles of *CES1* vary in the subethnic groups of Russia. The G allele frequency ranged from 22.5%–40.3% for European subethnic groups and those for Mongoloids, such as Buryats, amounted to 62.3%. Moreover, the frequencies of *CES1* rs2244613 and rs8192935 alleles are approximately 19.8% for the Finnish population (17).

Studies on the distribution frequency of the alleles and genotypes of *CES1* and *ABCB1* polymorphisms involved in anticoagulant metabolism have not been conducted among patients with AF in the Kazakh subpopulation. The significance of the impact of pharmacogenetic factors on the risk of anticoagulant therapy, and the possibility of optimizing anticoagulant therapy among individuals with AF in the Kazakh subpopulation, have not been studied. 

Accordingly, we investigated the association of enzymes encoding gene polymorphisms and proteins involved in the metabolism of new oral anticoagulants with the pharmacokinetics of dabigatran etexilate in patients with non-valvular AF in the Kazakh subpopulation. Further, we assessed the development of personalized solutions aimed at minimizing risks in real-life conditions and medical practice, which represents the actual direction of scientific research. We used the Kazakh subpopulation as our study subject because of the interracial and interethnic differences in the sensitivity of individuals to oral anticoagulants and the fact that there are no existing studies on this population.

## Methods

**Study participants:** The study participants were men and women aged 18–75 years. The median age was 58.9 years. The study group consisted of 150 Kazakh patients with non-valvular AF. Only volunteers were included in the study. Patient safety procedures were strictly adhered to according to the requirements of the Joint Commission International. This study was approved by the Ethics Committee of the National Scientific Cardiac Surgery Center in Astana, Kazakhstan (No. 01-74, dated June 10, 2021). All patients were familiar with the nature of the study — a researcher explained the essence, benefits, and risks of the study in detail. With a positive decision, each patient signed an informed consent form before participating in the study. Factors that may have affected plasma drug concentrations are listed in the exclusion criteria. The exclusion criteria were: 1) valvular AF; 2) the presence of thrombosis in the left atrial appendage according to transesophageal echocardiography; 3) a history of peptic ulcers or clinically active bleeding; 4) severe liver failure or renal dysfunction; 5) cancer; 6) allergic reaction to dabigatran; 7) co-administration of potent P-gp inhibitors (systemic ketoconazole, cyclosporine, and mus); 8) oncological diseases; and 9) refusal to participate in the study.

**Study design:** This was a clinical, prospective, observational, and controlled study. Patients were selected from those admitted to the National Research Cardiac Surgery Center between August 2021 and July 2022. Laboratory and instrumental tests were performed in accordance with standard clinical practice. The risk factors for stroke were assessed using the CHA2DS2-VASc scale for the appointment of direct oral anticoagulants for all patients. All patients were prescribed 150 mg dabigatran (Pradaxa, Boehringer-Ingelheim, Germany) twice daily in accordance with the clinical protocol of the Ministry of Health of the Republic of Kazakhstan.

**Study drugs:** This study was strictly conducted according to the approved scheme on an AST-TOP 500 analyzer (Instrumentation Laboratory, San Diego, CA, USA) with original sets of reagents from the same company. Mandatory quality control was performed daily; calibrations were performed when introducing the research methods and changing reagents. To determine the dabigatran concentration, citrated plasma was centrifuged at 3,000 rpm for 10 min.

 Venous blood samples were collected 1–3 h after the administration of dabigatran to measure the peak concentration. Subsequently, blood was collected again after 10–11 h to measure the minimum concentration. Upon admission, the patients were examined using a standard set of coagulograms (INR, APTT, PT, and PI).

**SNP genotyping:** DNA was isolated from venous whole blood samples using a GeneJET Whole Blood Genomic DNA Purification Mini Kit (Thermo Fisher Scientific, Waltham, MA, USA). The SNP genotyping was performed using Custom TaqMan SNP genotyping assays. The DNA was quantitatively analyzed using a NanoDrop 1000 spectrophotometer (Thermo Fisher Scientific). 

Genotyping for *ABCB1* (rs4148738, rs1045642, rs2032582, and rs1128503) and *CES1* (rs8192935, rs2244613, and rs71647871) SNPs was performed using Custom TaqMan SNP genotyping assays (https://www.thermofisher.com/order/genome-database/details/genotyping).

**Statistical analysis:** The independent impact of genetic polymorphisms on plasma dabigatran concentrations was analyzed via one-way analysis of variance (ANOVA). All statistical tests were two-sided. A significance level (α) of 0.05 was chosen for descriptive statistics. Following the Bonferroni correction or multiple comparisons, P = 0.007 was used to assess the significance level of associations between the SNPs and PDC.

The study was conducted in accordance with the Declaration of Helsinki and approved by the Ethics Committee of the National Research Cardiac Surgery Center (Protocol #01-74, from June 10, 2020). 

## Results

A total of 150 individuals participated in this study. The median age was 58.9 years. The anthropometric and biochemical characteristics of the study patients are presented in table 1. 

According to genotypic data, the distribution frequency of genotypes by loci of the *ABCB1* rs4148738 gene was as follows: CC – 32 patients (21.3%), CT – 64 (42.7%), and TT – 54 (36%); the minor allele frequency (MAF) was 40.4%. According to SNP rs1045642 of the *ABCB1* gene, 33 patients were AA genotype carriers (22%), 70 were AG carriers (46.7%), and 47 were GG carriers (31.3%); the MAF was 38.3%. Fifty-three patients (35.3%) were carriers of the CC genotype of the rs2032582 *ABCB1 *SNP, whereas 52 (34.7%) and 45 (30%) were carriers of the CA and AA genotypes, respectively; the MAF was 56.6%. For the rs1128503 *ABCB1 *SNP among the patients, 53 (35.3%) had the AA genotype, 62 (41.3%) had the AG genotype, and 35 (23.3%) had the GG genotype; the MAF was 62.6%. For *CES1*, the allele frequency distribution for SNP rs8192935 was as follows: 56 patients were AA genotype carriers (37.3%), 75 were AG carriers (50%), and 19 were GG carriers (12.7%); the MAF was 61.2%. The allele frequencies for the rs2244613 *CES1 *SNP were as follows: 37 (24.7%) patients had the GG genotype; 70 (46.7%) had the GT genotype, and 43 (28.7%) had the TT genotype; the MAF was 57.1%. 

For the rs71647871 *CES1 *SNP, the distribution was as follows: 148 (98.7%) patients had the GG genotype, 2 (1.3%) had the AG genotype, and none of the examined patients were carriers of the AA genotype; the MAF was 0% (table 2).

The distribution of all studied genotypes was assessed for Hardy–Weinberg correspondence using the χ^2^ test. The results were consistent with the Hardy–Weinberg law at p >0.05. The two polymorphisms, rs2032582 and rs71647871, were not in Hardy–Weinberg equilibrium and were excluded from subsequent analysis.

**Figure 1:**
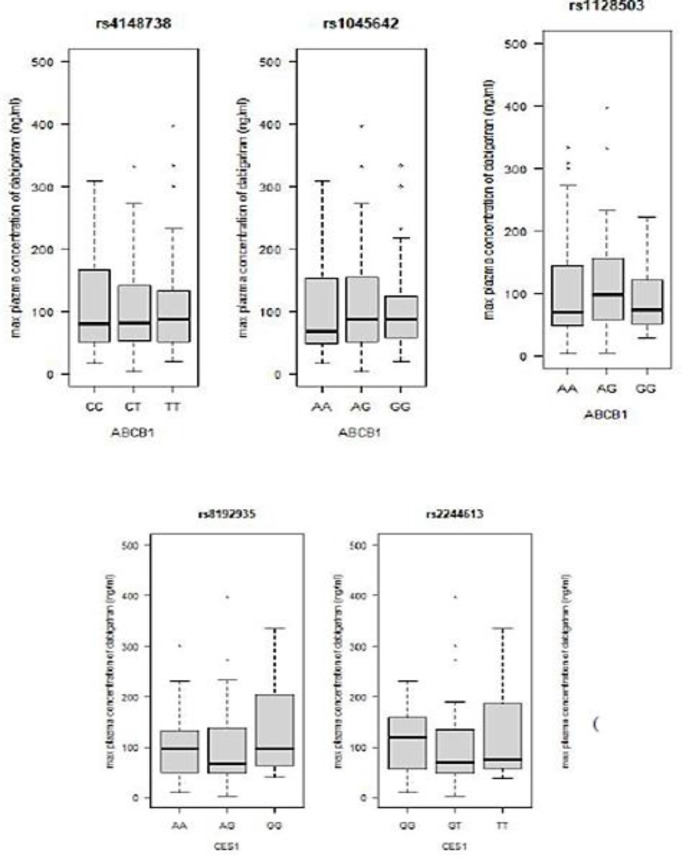
Maximum (A) and minimum (B) plasma concentrations of dabigatran with regard to the different genotypes of ABCB1 and CES1.

The distribution of the maximum and minimum dabigatran concentrations for the various *ABCB1* and *CES1* genotypes is displayed in figure 1. The PDCs among different genotypes were collated using ANOVA (table 3).

**Table 1:** Demographics and clinical features of the enrolled patients

**Variables**	**Characteristics of the study population** **(n = 150)**
**Age (years), mean (SD)**	58.9 (10.3%)
**Male, n (%)**	90 (60%)
**BMI (kg/m** ^2^ **), mean (SD)**	27.8 (3.5%)
eGFR (mL/(min*1.73 m ^2^ **)), mean (SD)**	_
CHA2DS2-VASc*, mean (SD)	1.55 (0.99%)

**Notes:** Quantitative variables are presented as the mean and standard deviation (SD). BMI, body mass index.*The CHA2DS2-VASc score (total 0–9 points) was determined by 1 point for each of the following: congestive heart failure/left ventricular dysfunction, hypertension, diabetes mellitus, vascular disease, female, aged 65–74; 2 points for each of the following: aged ≥75, previous stroke/transient ischemic attack/thromboembolism.

**Table 2:** Distribution of the ABCB1 and CES1 genotypes and allele frequencies in our test population (n = 150)

**Gene**	**SNP**	**Genotype**	**N (%)**	**Minor allele**	**MAF (%)**	**MAF (Global1000G)**	**HWE ** **P-value**
***ABCB1***	rs4148738	CC CT TT	32 (21.3) 64 (42.7) 54 (36)	C	40.4	38.14	0.149
***ABCB1***	rs1045642	AA AG GG	33 (22) 70 (46.7) 47 (31.3)	A	38.3	39.52	0.553
***ABCB1***	rs2032582	CC CA AA	53 (35.3) 52 (34.7) 45 (30)	A	56.3	33.43	0.0003
***ABCB1***	rs1128503	AA AG GG	53 (35.3) 62 (41.3) 35 (23.3)	A	62.6	41.61	0.064
***CES1***	rs8192935	AA AG GG	56 (37.3) 75 (50) 19 (12.7)	A	61.2	58,05	0.508
***CES1***	rs2244613	GG GT TT	37 (24.7) 70 (46.7) 43 (28.7)	G	57.1	33.27	0.498
***CES1***	rs71647871	AA AG GG	0 2 (1.3) 148 (98.7)	A	0	No frequencies available	1.429e-09

**Notes:** Values are presented as numbers (N) or percentages.

**Abbreviations:** SNP, single-nucleotide polymorphism; MAF, minor allele frequency; HWE, Hardy–Weinberg equilibrium.

In our study, the maximum and minimum plasma concentrations of dabigatran were compared between different genotypes. Genetic variants of CES1 (rs8192935 and rs8192935) were significantly associated with minimal dabigatran concentrations in the additive and recessive models.

Patients with the rs2244613 GG genotype had lower concentrations than those with the AA genotype (P = 0.000). Individuals with the rs8192935 AA genotype had lower concentrations than those with the GG genotype (P = 0.001).

In our study, clotting factors, including APTT at peak PDC, were analyzed among the different genotypes (table 4). We observed that the *ABCB1* SNPs, rs4148738 and rs1045642, were related to APTT at a minimal level, consistent with their effect on the dabigatran trough levels. The APTT values among the different genotypes of the *ABCB1* SNPs, rs4148738 and rs1045642, were significantly different (P = 0.035 and P = 0.024, respectively; table 4). Carriers of the TT genotype of SNP *ABCB1* rs4148738 had elevated APTT values compared to those of the CC genotype carriers (P = 0.012), and another post hoc pairwise matching indicated no statistical significance (CT vs. CC, P = 0.286; TT vs. CC, P = 0.144; TT vs. CT, P = 0.867). 

**Table 3:** Association of the SNPs with plasma concentrations of dabigatran

	**Plasma concentrations of dabigatran_Max**		**Plasma concentrations of dabigatran_MIN**	
	**Mean ± SD**	**P-value**	**Mean ± SD**	**P-value**
**rs8192935**				
Additive		0.016		**0.001**
AA	100.95 ± 59.64		52.72 ± 30.45	
AG	98.71 ± 72.32		51.61 ± 35.12	
GG	138.79 ± 100.15		79.78 ± 57.48	
Dominant		0.020		0.012
AA	100.95 ± 59.64		52.72 ± 30.45	
AG + GG	106.72 ± 79.71		57.24 ± 41.77	
Recessive		0.014		**0.000**
AA + AG	99.65 ± 67.05		52.08 ± 33.12	
GG	138.79 ± 100.15		79.78 ± 57.48	
Allelic		0.002		**0.000**
A	100.03 ± 64.79		52.27 ± 32.28	
G	112.07 ± 83.83		61 ± 45.27	
**rs2244613**				
Additive		0.066		**0.000**
GG	110.94 ± 61.32		55.27 ± 34.22	
GT	94.57 ± 68.26		50.82 ± 27.41	
TT	115.03 ± 86.94		63.33 ± 52.25	
Dominant		0.121		0.324
GG	110.94 ± 61.32		55.27 ± 34.22	
GT + TT	102.53 ± 76.36		55.69 ± 39.27	
Recessive		0.028		**0.000**
GG + GT	100.28 ± 66.09		52.37 ± 29.87	
TT	115.03 ± 86.94		63.33 ± 52.25	
Allelic		0.014		**0.000**
G	103.04 ± 64.85		53.12 ± 30.96	
T	106.04 ± 79.38		57.83 ± 43.26	
**rs4148738**				
Additive		0.616		0.175
CC	109.18 ± 74.98		60.54 ± 37.83	
CT	99.55 ± 68.41		53.72 ± 33.71	
TT	108.05 ± 77.67		54.88 ± 43.13	
Dominant		0.815		0.967
CC	109.18 ± 74.98		60.54 ± 37.83	
CT + TT	103.37 ± 72.52		54.24 ± 38.06	
Recessive		0.417		0.085
CC + CT	102.72 ± 70.4		55.97 ± 35.07	
TT	108.05 ± 77.67		54.88 ± 43.13	
Allelic		0.662		0.214
C	104.33 ± 71.32		57.11 ± 35.68	
T	104.82 ± 73.96		54.44 ± 39.57	
**rs1045642**				
Additive		0.549		0.214
AA	101.03 ± 73.75		54.65 ± 39.16	
AG	107.26 ± 77.18		56.91 ± 34.17	
GG	103.08 ± 66.4		54.2 ± 43.15	
Dominant		0.932		0.802
AA	101.03 ± 73.75		54.65 ± 39.16	
AG + GG	105.62 ± 72.86		55.85 ± 37.8	
Recessive		0.305		0.124
AA + AG	105.28 ± 75.81		56.2 ± 35.65	
GG	103.08 ± 66.4		54.2 ± 43.15	
Allelic		0.488		0.358
A	104.26 ± 75.07		55.82 ± 36.39	
G	104.9 ± 70.9		55.38 ± 39.25	
**rs1128503**				
Additive		0.010		0.149
AA	104.35 ± 81.85		57,82 ± 42.21	
AG	114.87 ± 74.97		59.23 ± 37.51	
GG	87.6 ± 50.15		46.11 ± 30.92	
Dominant		0.119		0.159
AA	104.35 ± 81.85		57.82 ± 42.21	
AG + GG	104.75 ± 67.86		54.36 ± 35.62	
Recessive		0.003		0.086
AA + AG	109.98 ± 78.07		58.58 ± 39.59	
GG	87.6 ± 50.15		46.11 ± 30.92	
Allelic		0.010		0.061
A	108.19 ± 79.08		58.34 ± 40.31	
G	100.11 ± 63.83		52.13 ± 34.49	
**rs71647871**				
Additive		0.854		0.387
AA				
AG	100.85 ± 62.41		50.64 ± 15.2	
GG	104.66 ± 73.15		55.65 ± 38.2	
Dominant				
AA				
AG + GG	104.61 ± 72.84		55.58 ± 37.97	
Recessive		0.854		0.387
AA + AG	100.85 ± 62.41		50.64 ± 15.2	
GG	104.66 ± 73.15		55.65 ± 38.2	
Allelic		0.857		0.388
A	100.85 ± 62.41		50.64 ± 15.2	
G	104.63 ± 72.87		55.62 ± 38.02	

**Table 4:** Comparison of the coagulation parameters with regard to different genotypes

**Gene**	**SNP**	**Genotype**	**aPTT** **at min level(s), mean (SD)**	**TT** **at min level(s), mean (SD)**
***ABCB1 *** **Kruskal–Wallis ** ***P-value*** **: **	rs4148738	CC CT TT	35.1 (6.7) 37.6 (6.4) 38.3 (9.4) P = 0.035	12.9 (2.8) 12.4 (3.3) 12.6 (3.1) P = 0.281
***ABCB1 *** **Kruskal–Wallis ** ***P-value*** **: **	rs1045642	AA AG GG	34.9 (6.2) 38.3 (6.8) 37.5 (9.5) P = 0.024	12.8 (2.8) 12.2 (2.5) 13.1 (3.9) P = 0.395
***ABCB1 *** **Kruskal–Wallis ** ***P-value*** **: **	rs1128503	AA AG GG	36.3 (7.2) 37.9 (6.2) 37.9 (10.4) P = 0.173	12.6 (2.3) 12.7 (3.8) 12.5 (2.7) P = 0.408
*CES1* **Kruskal–Wallis ** ***P-value*** **:**	rs8192935	AA AG GG	38.3 (3.4) 36.7 (7.6) 36.7 (5.8) P = 0.750	12.3 (2.3) 12.9 (3.8) 12.1 (1.2) P = 0.829
*CES1* **Kruskal–Wallis ** ***P-value*** **:**	rs2244613	GG GT TT	36.9 (10.2) 38.1 (7.4) 36.4 (5.5) P = 0.559	12.3 (2.0) 12.7 (3.8) 12.7 (2.6) P = 0.693

## Discussion

Initially, this study was conducted to determine the distribution frequency of genotypes and alleles of the polymorphisms in *ABCB1* and *CES1* involved in the metabolism of dabigatran. It is necessary to conduct replicative studies because the distribution frequency of alleles and genotypes varies greatly in different populations. Our findings suggested that allele distribution frequencies of the *ABCB1* and *CES1* polymorphisms in the Kazakh subpopulation were similar to those recorded in Asian and East Asian populations. For example, allele frequencies of the rs4148738 and rs1045642 polymorphisms of *ABCB1* were comparable to those in the general population (1,000 genomes). However, the occurrence frequency of the A allele (62.3%) of rs1128503 in the studied population differed from that in the general population (41.61%). At the same time, the occurrence frequency of the A allele of rs1128503 coincided with that of East Asian populations (62.7%) and differed significantly from that of African populations. The frequency of allele A (61.2%) of *CES1* rs8192935 was, in principle, comparable to that in the general population (58.05%). However, the frequency of allele G (57.1%) of *CES1* rs2244613 in the Kazakh subpopulation differed significantly from that in the general population (33.27%). This occurrence frequency is similar to that of East Asian populations but differs significantly from that of European populations, exceeding the frequency by 3.5 times. Moreover, the frequency of the G allele in the Kazakh subpopulation was higher than that in African (24.89%) and American populations (27.2%).

Sychev et al. (16) reported that the occurrence frequency of the peaceful allele rs2244613 of *CES1* in Siberian and far eastern Buryats was as high as that in the Kazakh subpopulation (62.3%). According to the results of similar studies (8), 10 carriers of the minor G allele of the rs2244613 gene polymorphism were statistically associated with a decrease in the amount of bleeding. Liu et al. (13) conducted research on a Chinese population and demonstrated that the allele frequencies of *CES1* rs2244613 and rs8192935 were inconsistent with those observed in Caucasians. Differences were observed when comparing the allele occurrence frequencies of these polymorphisms between the Chinese population and Kazakh subpopulation — the frequency was higher in the Kazakh subpopulation. Li et al. (18) conducted a meta-analysis and reported that patients carrying at least one minor allele G (*CES1* rs2244613) were associated with reduced dabigatran levels and a lower risk of bleeding than in the non-carriers (i.e., T/T genotype). Given the results obtained for the Kazakh subpopulation (high frequency of the minor G allele), we speculate that dabigatran administration poses a lower risk of bleeding in patients. Regarding the *ABCB1* rs1128503 polymorphism, no significant correlation was observed between locus variations and cases of bleeding in previous studies. Consistent with existing literature, our results revealed a significant association between the minimum plasma concentration of dabigatran and the rs2244613 and rs8192935 polymorphism genotypes of *CES1* of the dabigatran residual steady-state concentration. *ABCB1* polymorphisms did not affect the pharmacokinetics of dabigatran.Liu et al. (13) reported that *CES1* rs8192935 was associated with the peak concentration of dabigatran in a Chinese population. Meanwhile, Roşian et al. (19) reported the lack of a statistically significant relationship between *CES1* or *ABCB1* polymorphisms and plasma levels of dabigatran; only a tendency toward a decrease in the minimum plasma level was observed in carriers of variant SNP rs8192935 alleles. Their study was conducted with a European population (genotype–phenotype correlation for dabigatran in patients with non-valvular AF (single-center research)).

 A direct correlation between the concentration of dabigatran in plasma and the severity of the anticoagulant effect is well-established; dabigatran prolongs the APTT. Here, we also examined the genetic influence on the APTT and TT values. We determined that APTT values among different genotypes of the *ABCB1* rs4148738 and rs1045642 SNPs were significantly different (P = 0.035 and P = 0.024, respectively). However, Ji et al. (14) observed that different genotypes of *CES1* (rs8192935 and rs2244613) were minimally associated with TT and APTT.

The results obtained for the Kazakh subpopulation, namely the high frequency of the minor allele G rs2244613 of *CES1*, suggests that dabigatran administration is associated with a lower risk of bleeding in patients. Our study results suggest that the *CES1* polymorphisms, rs8192935 and rs2244613, are correlated with the pharmacodynamics and pharmacokinetics of dabigatran in the Kazakh subpopulation.
